# Sequential Notch Signalling at the Boundary of Fringe Expressing and Non-Expressing Cells

**DOI:** 10.1371/journal.pone.0049007

**Published:** 2012-11-12

**Authors:** Tobias Troost, Thomas Klein

**Affiliations:** Institut für Genetik, Heinrich-Heine-Universität Düsseldorf, Universitätsstr.1, Duesseldorf, Germany; National Institutes of Health (NIH), United States of America

## Abstract

Wing development in *Drosophila* requires the activation of Wingless (Wg) in a small stripe along the boundary of Fringe (Fng) expressing and non-expressing cells (FB), which coincides with the dorso-ventral (D/V) boundary of the wing imaginal disc. The expression of Wg is induced by interactions between dorsal and ventral cells mediated by the Notch signalling pathway. It appears that mutual signalling from dorsal to ventral and ventral to dorsal cells by the Notch ligands Serrate (Ser) and Delta (Dl) respectively establishes a symmetric domain of Wg that straddles the D/V boundary. The directional signalling of these ligands requires the modification of Notch in dorsal cells by the glycosyltransferase Fng and is based on the restricted expression of the ligands with Ser expression to the dorsal and that of Dl to the ventral side of the wing anlage. In order to further investigate the mechanism of Notch signalling at the FB, we analysed the function of Fng, Ser and Dl during wing development at an ectopic FB and at the D/V boundary. We find that Notch signalling is initiated in an asymmetric fashion on only one side of the FB. During this initial asymmetric phase, only one ligand is required, with Ser initiating Notch-signalling at the D/V and Dl at the ectopic FB. Furthermore, our analysis suggests that Fng has also a positive effect on Ser signalling. Because of these additional properties, differential expression of the ligands, which has been a prerequisite to restrict Notch activation to the FB in the current model, is not required to restrict Notch signalling to the FB.

## Introduction

The Notch signalling pathway is an evolutionary conserved short-range signalling pathway that is involved in numerous developmental processes and diseases [1]–[3]. The pathway consists of three core elements, a DSL (Delta/Serrate/Lag 2) ligand, the Notch receptor itself and a transcription factor of the CSL (CBF1/Su(H)/Lag1) family. In *Drosophila* two ligands, Delta (Dl) and Serrate (Ser), exist together with one DNA binding CSL factor, Suppressor of Hairless (Su(H)). The binding of a ligand to Notch elicits a proteolytic cascade that results in the release of the intracellular domain of Notch (NICD). The cleavages are mediated by the metalloprotease Kuzbanian (Kuz)/ADAM10 and the γ-secretase complex with Presenilin (Psn) at its catalytic centre [4]–[6]. The released NICD associates with Su(H) and activates the transcription of the target genes. In the absence of Notch signalling Su(H) is part of a repressor complex that silences the target genes. An important factor in this complex is Hairless (H), which connects the co-repressors Groucho and CtBP with Su(H) [7]. Consequently, loss of *H* or *Su(H)* function results in the weak de-repression of several target genes in the absence of Notch signalling in *Drosophila* [8]–[10].

During *Drosophila* wing development, the Notch pathway mediates interactions between cells across the dorso-ventral (D/V) compartment boundary that activate and maintain the expression of several genes essential for the establishment, growth and patterning of the wing primordium, notably *wingless* (*wg*) and *vestigial* (*vg*) [11]. As a result of these interactions, the genes are eventually expressed in cells at both sides of the D/V boundary. This symmetrical expression at the D/V boundary is the result of signalling of Dl and Ser in opposite direction [12]–[14] and involves two regulatory loops. These loops operate at different times and depend on different expression patterns of the two Notch ligands during the third larval instar stage [12], [14]–[16]. During early stages, Ser is expressed in all dorsal boundary cells (DBCs) [17], [18], whereas Dl appears to be up-regulated in cells at the ventral side of the boundary (in ventral boundary cells (VBCs) [12], [15]. During this phase, the high expression of both ligands at the boundary appears to be maintained by the activity of the Notch pathway. This notion is based on the observation that ectopic activation of the pathway leads to ectopic expression of the ligands [14], [15], [19]. During this time the expression of *Ser* appears to be restricted to the dorsal and that of Dl is up-regulated at the ventral side of the wing anlage. Their expression is maintained by Notch activity induced through the initial regulatory loop (Dl/Ser loop) at these sides [14]. The differential expression of the ligands entails that the activation of Notch stays restricted to the D/V boundary and does not spread from the D/V boundary into the nascent wing pouch [14].

The mutual directional signalling of the ligands requires the activity of the glycosyltransferase Fringe (Fng) [19]–[21]. Fng modifies several EGF modules in the extra-cellular domain of Notch [22], [23]. This glycosylation regulates the interaction of Notch with its ligands, such that it enhances Dl- and suppresses Ser-signalling during the early phase of signalling. The expression of Fng and Ser is activated by the selector protein for the dorsal fate, Apterous (Ap) during early stages of wing development [17], [18], [20]. Hence, the boundary of Ser- and Fng expressing and non-expressing cells coincides with the D/V boundary. Due to the presence of Fng in all cells that express Ser, Ser can activate Notch only in adjacent VBCs, which produce unmodified Notch. In contrast Dl, signals preferentially to DBCs, where Notch is modified. This polarization of signalling results in the symmetrical activation of Notch in DBCs and VBCs [24], [25]. Hence, it is the boundary of Fng expressing and non-expressing cells (Fringe-boundary, FB) that regulates the activation of Notch signalling at the D/V boundary. It has to be noted here that the expression pattern of Dl during early stages is difficult to determine and it has never been tested whether the weak staining observed throughout the early third instar disc is specific.

After establishment of the Wg expression domain, a second regulatory loop is initiated and maintains the activity of Notch in boundary cells [12], [16]. The expression of Dl and Ser is now controlled by Wg (Dl/Ser/Wg loop). Wg, secreted by boundary cells, diffuses to adjacent dorsal and ventral pouch cells where it induces the expression of both ligands in a narrow dorsal and ventral band next to the Wg domain. [12], [16]. Dl and Ser are thought to signal back from these cells to the boundary cells to maintain expression of Wg [12], [16]. At this later phase, the expression pattern of Fng changes [20] and its contribution to the second loop has not been investigated.

The current model suggests that Notch signalling at the D/V boundary respectively FB is symmetric throughout wing development [26]. However, this assumption has not been investigated rigorously. Indeed, some observations suggest that signalling is initially asymmetric [16]. Moreover, Notch signalling is also required for the establishment and maintenance of the D/V compartment boundary. Consequently, loss of Notch signalling in boundary cells results in the loss of the compartment boundary and mutant clones cross over into the other compartment [27], [28]. Thus, it is difficult to unambiguously determine the compartmental origin of the clone, which is a prerequisite to determine the spatial requirements of components of the Notch pathway such as the ligands during signalling at this boundary using mosaic analysis. This might be a reason for the partial differences in the results obtained by the different groups [13], [15]–[17]. The results of Couso et al. [17] and deCelis et al [13] indicate that Ser is required in DBCs, suggesting it mediates signalling from DBCs to VBCs. Miccheli et al. [16] reported cell clones mutant for each of the ligands occasionally induced loss of target genes. However, this loss was not reliable. Moreover, deCelis et al. [13] and Doherty et al. [15] reported that only Dl clones that contained dorsal and ventral cells caused extensive scalloping of the wing, while clones that abutted the ventral side of the boundary caused weak scalloping and dorsal clones had no effect. However, the later two groups analysed adult wings and it is not clear when during development the wing tissue is lost. In contrast Micccheli et al. analysed imaginal discs. This might account for the differences. In addition, the clonal analysis so far does not strictly take into account the existence of the two different feedback-loops and the importance of the ligand therein. The importance of each ligand might be different in the two regulatory loops.

In order to further investigate the mechanism of Notch signalling at a FB, we analysed the function of Fng, Ser and Dl during early stages of wing development at an ectopic FB. With help of this information, we re-examined the situation at the D/V boundary. We find that Notch signalling is initiated in a sequential fashion at both boundaries. During an initial asymmetric phase, only one ligand is required. The ligand required as well as the location of Notch activation relative to the *Fng* domain differs.

## Results

### Asymmetric Expression of Notch Target Genes at an Ectopic FB

**Figure 1:**
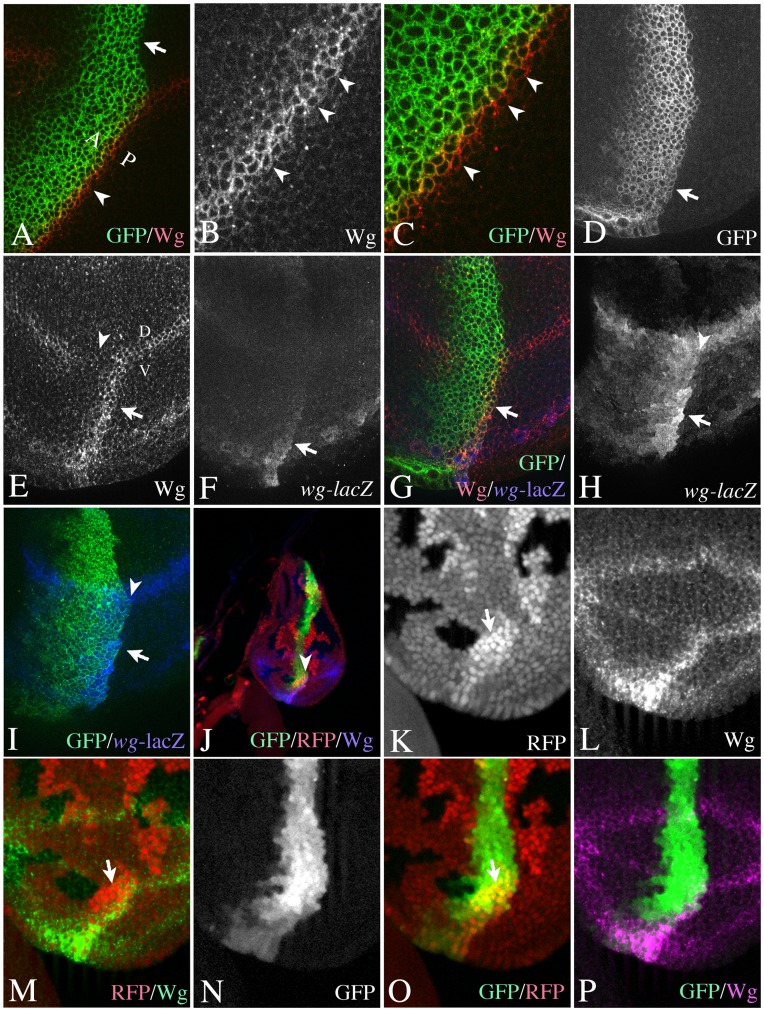
Asymmetric Notch-signalling at the ectopic FB during early wing development. (A–C) Ectopic expression of Wg in late third instar discs occurs in boundary cells at both sides of the boundary. Arrowheads highlight the expression of Wg in PBCs. The expression domain of *ptc*Gal4 revealed by fluorescence of UAS GFP-GPi. Note, that *ptc*Gal4 expression increases towards the A/P boundary and is highest in ABCs. It is not expressed in PBCs. (D–I) Ectopic activation of expression of Wg and *wg*-lacZ upon expression of UAS *fng* with *ptc*Gal4 in early third instar wing imaginal discs. (D) The expression domain of *ptc*Gal4 revealed by fluorescence of UAS GFP-GPi. (E–I) The induced ectopic expression of Wg (arrow in E) and *wg*-lacZ (F) is restricted to the GFP-positive Fng expressing ABCs (G, I). The arrowhead highlights the area where the expression of Wg along the D/V boundary is interrupted because of the ectopic expression of Fng. (H, I) The same disc as in (D–G) focussed on the expression of *wg*-lacZ. The merge shown in (I) reveals that the expression of *wg*-lacZ is restricted to anterior Fng expressing cells. Arrowhead in (H, I) highlight the A/B boundary. (J–P) Mapping of the ectopic expression domain of Wg using a clonal analysis. The A/P boundary was additionally revealed through the expression of UAS GFP, which labels the *ptc* expression domain. The arrowhead in (J) highlights a region containing RFP-homozygous clone abutting the anterior side of the A/P boundary, which is shown in (K-P) at higher magnification. The arrows in (K, M, O) point to the RFP homozygous clone.

In order to generate an ectopic FB where the spatial requirement of Dl and Ser can be investigated, we ectopically expressed UAS *fng* with *ptc*Gal4. *ptc*Gal4 activates expression of UAS *fng* in a broad stripe of cells at the anterior side of the antero-posterior compartment boundary (A/P boundary) with increasing expression towards the posterior (see Fig. 1A, D and Fig. S1A–C). If expressed in this way, the sharp posterior expression boundary of UAS *fng* coincides with the antero-posterior (A/P) compartment boundary (arrow in Fig. 1A, D). Expression of Wg at the beginning of third larval instar stage is initially expressed throughout the ventral part of the wing imaginal disc (Fig. S1A, A’). The expression resolves in a domain along the D/V boundary and a ring-like domain, which surrounds the wing anlage (Fig. S1B, B’). The domain at the D/V boundary is induced by Notch signalling and is required for wing development. During later stages a second ring-like domain appears (Fig. S1C, C’). The ectopic expression of Fng results in a stripe of ectopic expression of Wg that straddles the sharp posterior FB in late third instar wing pouches in a similar manner as at the D/V-boundary ([29]; Fig. 1A–C). This symmetric expression of Wg is restricted to ventral pouch cells (arrowhead in Fig. 1A, arrow in Fig. 1E–I), since endogenous expression of Fng in all dorsal cells prevents the formation of a strong FB there.

We initially focussed on wing discs of the early/mid third instar stage and monitored the expression of Wg and *wg*-lacZ as read-out for the activity of Notch. We found that during these stages, the expression of both markers map to cells of the anterior side of the A/P boundary (anterior boundary cells, ABCs), which express Fng (Fig. 1D–I).

Cells of the anterior and posterior compartments never cross the A/P boundary. Instead they align along the boundary to form a straight border that can be visualised by lineage labelling techniques such as clonal analysis. Using clonal analysis, we confirmed that the ectopic stripe of Wg expression mapped to Fng expressing ABCs (Fig. 1J–P). These results suggest that Notch is activated asymmetrically at the ectopic FB only in Fng expressing ABCs during early stages of wing development, while it is activated symmetrically at both sides of the ectopic FB in late stages.

In late third instar discs, the expression of Dl at the ectopic FB occurs in two stripe-like domains adjacent to the Wg expressing cells, indicating that the later operating feedback loop is established in a similar manner than at the D/V boundary (Fig. S1D–F).

### Requirement of Notch Activity at the Ectopic FB

**Figure 2:**
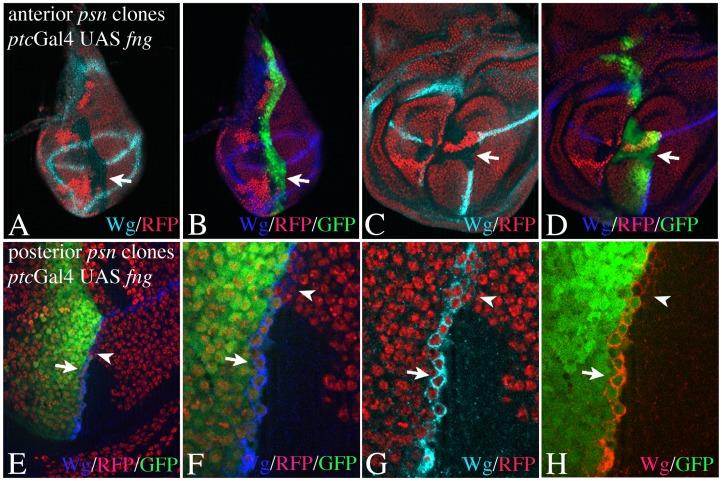
Spatial requirement of Notch activity at the ectopic FB at early and late stages of wing development. Clones are labelled by the absence of the GFP or RFP marker. (A–D) *psn* clones abutting the anterior side of the A/P boundary result in a loss of Wg expression in early (A, B) and late discs (C; D). (E–F) A *psn* clone abutting the posterior side of the boundary in a disc at late stages. The expression domain remains to be restricted to wildtype ABCs (arrows) in the region of the clone. In regions of wildtype PBCs the epression of Wg is symmetric and occurs in ABCs and PBCs (arrowheads).

To further confirm the asymmetry in Notch activation, we determined on which side of the ectopic FB the activity of Notch is required by inducing clones mutant for *psn* abutting on each side of the boundary (Fig. 2). In contrast to the D/V boundary, establishment and maintenance of the A/P boundary is not dependent on the activity of the *Notch* pathway and clones mutant for genes encoding members of the pathway respect this boundary [27], [28]. We co-expressed UAS GFP to label the A/P boundary and ABCs in the *ptc* domain. This enabled us to unambiguously determine the origin of the clone cells at the A/P boundary. We found that only *Psn* mutant clones abutting the anterior side of the A/P boundary, abolished expression of Wg at the ectopic FB at early stages (Fig. 2A, B) and late stages (Fig. 2C, D). This result confirms that Notch is asymmetrically activated in ABCs. It further indicates that the activation of Notch in ABCs at early stages is a prerequisite for the establishment of the late symmetric expression of Wg. Clones abutting the posterior side of the ectopic FB do not affect expression of Wg in ABCs, but prevents the establishment of the late symmetric expression in ABCs (Fig. 2E–H). This is indicated by the lack of its expression in mutant posterior boundary cells (PBCs) in discs in late stages (Fig. 2E–H). Thus, activation of Notch in PBCs is required for expression of Wg during the late symmetric phase of expression. The results also suggest that a Dl/Ser feedback loop established in boundary cells is not important during the early asymmetric phase to induce Wg expression at the ectopic FB.

### Requirement of Dl and Ser at the Ectopic FB

**Figure 3:**
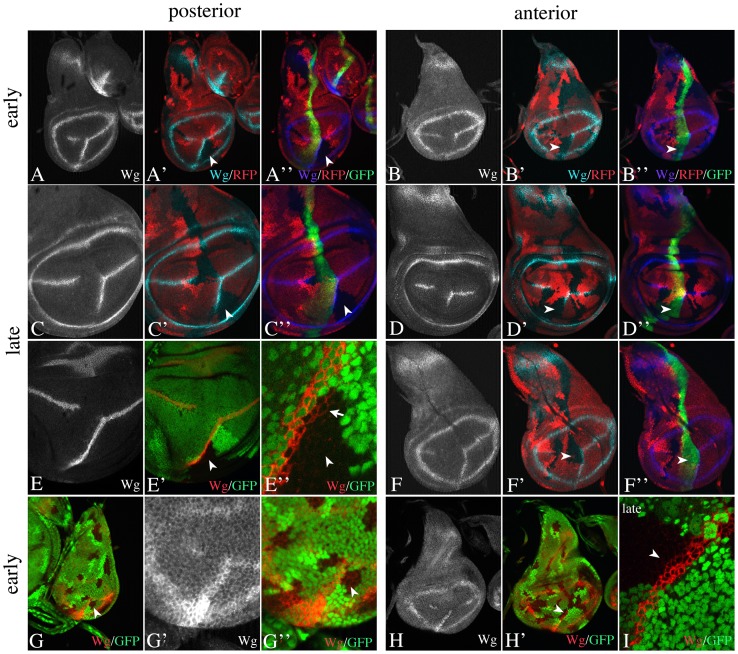
Spatial requirement of Ser activity at the ectopic FB during wing development. Clones are labelled by the absence of the GFP or RFP marker. (A, C, E, G) *Ser* clones abutting the posterior side of the A/P boundary do not affect the expression of Wg in early (A, G) as well as in late (C, E) stages. The arrowheads highlight the clone abutting the posterior side. (E) A disc in the late stages of third larval instar bearing a large *Ser* clone abutting the posterior boundary. (E’’) Magnification of the region highlighted in (E’) by the arrowhead. It reveals that the symmetric late phase of Wg expression is normally established indicated by the expression of Wg in the mutant territories in late stages (arrow in E’’). (B, D, F, H) Ser clones abutting the anterior side had variable effects. Arrowheads highlight the clones. A fraction of discs showed a strong shortening of the ectopic expression domain of Wg in the mutant area (B, D). In other cases the expression appears to be unaffected (F, H). (I) In these cases the expression in late stages remains restricted to ABCs. Thus, the symmetric phase is not established in the mutant region.

**Figure 4:**
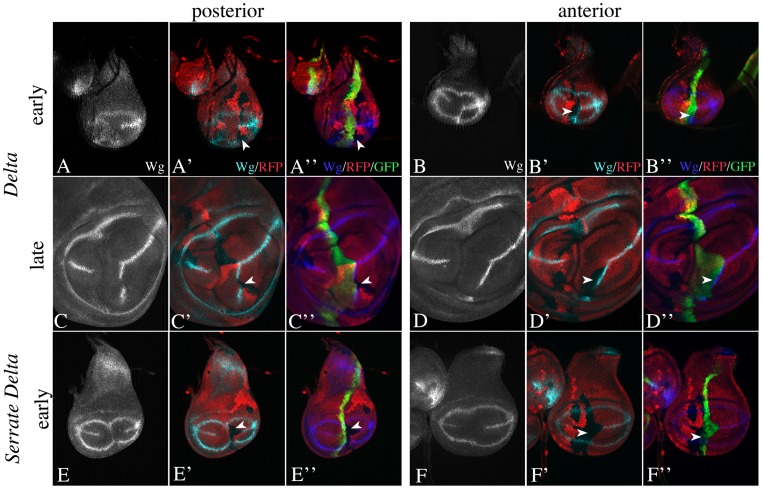
Spatial requirement of Dl activity at the ectopic FB during wing development. (A–D) Dl clones abutting the posterior (A, C) and anterior (B, D) side of the A/P boundary. (A, C) Only clones abutting the posterior side (arrowheads) abolish the expression of Wg, indicating that Dl is required in PBCs. The expression of Wg is not recovered in later stages (C). Anterior clones do not exhibit any effect on Wg expression. (E, F) *Dl Ser* double mutant clones abutting the posterior (E) and anterior (F) side of the boundary abolish expression of Wg already during early stages.

In order to determine which ligand is required for the induction of the early asymmetric expression of Wg at the ectopic FB, we monitored *Ser-* and *Dl*-mutant clones abutting the A/P boundary (Fig. 3, 4). Please take in account that we observed that the ectopic domain of Wg expression at the end of the third larval instar is often shorter in *Dl* or *Ser*, but not *Psn* heterozygous wing discs (see Fig. S2). This effect is most pronounced for *Dl*. However, during early phases of the third instar stage, the ectopic induction of Wg expression is undistinguishable in all three genotypes and reaches until the ring-like domain of Wg expression (Fig. S2). In an initial round of experiments, we induced clones without revealing the A/P boundary through co-expression of UAS *GFP*. However, in many cases we unable to unambiguously determine the compartmental origin of the clones. Thus, we co-expressed UAS *GFP* in the subsequent experiments. Nevertheless, we included some of the clones of the initial experiments where the origin is clear in our analysis.

We found that loss of *Ser* function in cells on the posterior side of the FB had little effect on *wg*-expression during early and late stages (Fig. 3A, C, E, G). Wg was expressed normally and symmetric expression was established (Fig. 3E). However, the loss on the anterior side had variable effects in early and late discs (Fig. 3B, D, F, H, I). In many discs, we observed a dramatic shortening of the ectopic domain of Wg expression in the clone regions (Fig. 3B, D). In other discs, the ectopic domain of Wg expression is present (Fig. 3F, H, I). In these cases, we never observed symmetric expression in late stages (Fig. 3I). These observations suggest that Ser contributes to activation of Wg expression in Fng expressing ABCs. They also indicate that it is required for the establishment of the symmetric expression of Wg in late stages. Thus Ser signals from ABCs to PBCs during late stages.

We found that *Dl*-clones abutting the posterior side of the boundary abolished the expression of Wg in adjacent ABCs in early discs (Fig. 4A, C). This indicates that a Dl-mediated signal from PBCs to ABCs is required to establish the early asymmetric Wg expression. Posterior *Dl*-clones also prevented expression of Wg normally observed in late third instar wing discs (Fig. 4C). This reaffirms the conclusion that the early asymmetric phase of Notch activation is a prerequisite for the induction of the later symmetric one. Anterior abutting clones had no obvious effect on expression of Wg (Fig. 4B, D).

Next, we tested the behaviour of *Dl Ser* double mutant clones in discs at early stages (Fig. 4E, F). As expected posterior abutting clones extinguished the expression of Wg in ABCs (Fig. 4E). However, anterior clones also completely extinguished the expression of Wg indicating that the activity of the Notch pathway is abolished (Fig. 4F). Anterior *Ser* clones dramatically reduced the expression of Wg in a fraction of discs, but never abolished it (see above). Hence, the result indicates that also Dl contributes to the activation of Notch within Fng expressing ABCs.

### Signalling at an Ectopic FB in Absence of *Ser* Function

Loss of *Ser* function results in the loss of most wing structures including the margin and the pouch underlining its important function in wing development [30]. We have previously observed residual Notch activity in *Ser^94c^/Ser^RX106^* mutant wing imaginal discs during early third larval instar [19]. However, it is not clear whether this allelic combination represented a complete loss of function situation. During the course of our studies, we have generated a recombinant chromosome that bears the molecularly characterized null allele *Ser^RX106^* and the Gbe+Su(H)-lacZ construct, which faithfully detects the activity of the *Notch* pathway in imaginal discs [31]. The recombination event appeared to remove a lethal second site mutation, since we observed that a small number of homozygous animals developed to the pharate adult stage. For a detailed description of the *Ser* null phenotype, see Text S1.

We found that Gbe+Su(H)-lacZ was expressed at the D/V boundary in wing imaginal discs of this null mutant during early stages of third larval instar (Fig. S3B–D, G–Q). Interestingly, the expression was restricted to *ap* expressing dorsal wing cells and appears to have spread over the whole dorsal wing anlage (Fig. S3P, Q, arrowheads). Thus, weak activity of Notch, probably induced by Dl, is present in the wing primordium even in the complete absence of *Ser* function. However, activation of Notch appears to be too weak to activate expression of Wg. The finding indicates that the expression of Dl is to some extent independent of *Ser* function in early third instar wing discs. Expression of Gbe+Su(H)-lacZ is lost during later stages of wing development in the *Ser* mutant wing discs. This finding confirms that Ser has an additional function in maintenance of the activity of Notch.

When we ectopically expressed Fng with *ptc*Gal4 in *Ser^RX106^* null mutants, we found that Gbe+Su(H)-lacZ, but not Wg was ectopically expressed in the ventral side of the remaining wing anlage (Fig. S3R, S, arrows). The expression was restricted to the *ptc*Gal4 domain (Fig. S3R, S, arrows). This indicates that, like at the D/V boundary, Notch is activated ectopically, but not sufficiently enough to induce expression of Wg. Note, that the expression of Gbe+Su(H) covers the whole *ptc* domain. In late third instar discs the expression of Gbe+Su(H)-lacZ was lost (Fig. S3T).

### Induction of Wing Development in the Absence of *Ser* Function

Hairless (H) and Su(H) are central members of the repressor complex that forms in absence of Notch signalling to silence the expression of target genes [32]. In the absence of *H* function the repressor complex fails to form and weak de-repression of some Notch target genes, among them the wing selector gene *vg*, can be observed [10]. However, expression of Wg is not induced. We previously found that, in *H ap* double mutants, this de-repression is sufficient for the formation of a wing pouch, which is absent in *ap* mutants [33]. Here, we found that the loss of *H* function enhances the residual activity of Notch observed in *Ser* mutants to a degree that wing development can continue (Fig. S4). *Ser^RX106^ H^E31^* double mutants developed to pharate adults, which had elaborated wings with margins, as well as halteres (Fig. S4A). In the corresponding wing discs, the expression of Gbe+Su(H)-lacZ and Wg along the DV boundary was restored, but restricted to *ap*-expressing DBCs, even in late third instar discs (Fig. S4B-E).

Expression of Gbe+Su(H)-lacZ was abolished in *ap; Ser H* triple mutant wing imaginal discs (Fig. S4F), indicating that activation of Notch in *Ser H* mutant dorsal cells is probably dependent on the function of an Ap induced FB. Furthermore, Dl was expressed in *Ser H* double mutant discs (Fig. S4G), suggesting that it is responsible for the activation of Notch in *Ser* mutant wing discs. Thus, although the Dl signal is too weak to activate Wg in *Ser* mutants, it is sufficient in the sensitised *Ser H* double-mutant background. These results on the one hand confirm that signalling of the two ligands at the D/V boundary is to some extent independent of each other. On the other hand they suggest that Ser is required to achieve strong activation of Notch in cells at the D/V boundary that cannot be generated by Dl alone.

We found that in *Ser H* double mutants, Wg expression at the ectopic FB is restricted to Fng expressing anterior cells, even in the late third instar stages (Fig. S4J-N). Hence, these results support the conclusion that Ser is required to establish the late symmetric phase of Notch activation at the ectopic FB. Note, that in contrast to *H* mutant discs, Wg and Gbe+Su(H) –lacZ are expressed throughout the *fng* domain in *H Ser* mutant discs. This suggests that Ser suppresses the activity of Notch throughout the *fng* domain during normal development.

### Delta is Expressed in All Cells of the Wing Anlage

**Figure 5:**
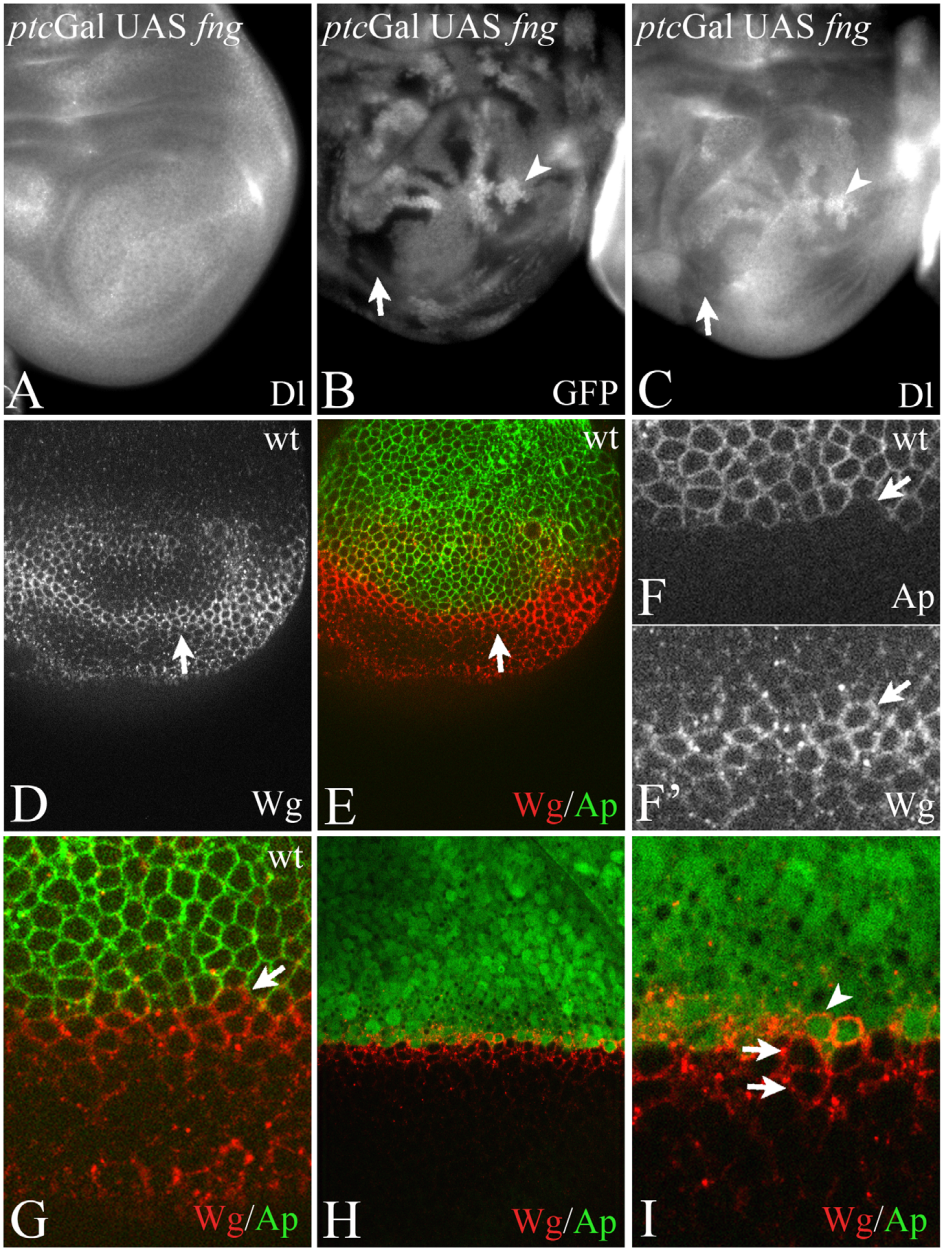
Dl is expressed throughout the wing primordium of early third instar wing imaginal discs. (A–C) Expression of Dl in an early third instar wing imaginal disc that express UAS *fng* with *ptc*Gal4. (A) Expression of Dl in a disc without clones. Expression in enhanced at the D/V boundary and the ectopic FB (arrow). (B, C) Expression of Dl in a disc bearing *Dl^rev10^* mutant clones. (B) The clones are labelled by the absence of GFP (arrowh in B, C). The arrowhead highlights a clone with cells homozygous for GFP and the Dl wildtype allele (GFP/GFP). (C) Expression of Dl in the same disc as in (B). The comparison of (B) and (C) reveals that the staining is strongly reduced in *Dl^rev10^* mutant clones and enhanced in cell clones homozygous for the wildtype allele. (D–I) Asymmetric Notch signalling at the D/V boundary. (D–G) Expression of Wg and Ap in an early third larval instar wing imaginal disc. (F, G) is a magnification of the D/V region of the disc shown in (D, E). The domain of Wg along the D/V boundary is largely restricted to ventral boundary cells adjacent to the Ap domain (see merge in D). (H, I) In late stages Wg is expressed also in DBCs. However, while it is restricted to DBCs (arrowhead in I), it is expressed in VBCs and the adjacent row of ventral cells (arrows in I).

The presented results indicate that Dl initiates Notch signalling at the ectopic FB. Thus, Dl must be expressed throughout the ventral wing pouch at early stages of wing development. To confirm this conclusion, we examined clones mutant for *Dl* in the wing anlage that were stained with an anti-Dl antibody (Fig. 5A–C). Previous work suggests that Dl is expressed at the D/V boundary, mainly in VBCs [12]. Additionally, diffuse weak expression is described either throughout the ventral or dorsal and ventral side [12], [15]. However, it has never been tested, whether this is specific or background produced by the staining procedure. We found that the diffuse signal was strongly reduced in *Dl* mutant clones (Fig. 5B, C arrow). Furthermore, cells of the wild-type twin clones, which contained two copies of *Dl*, had higher levels of staining than their heterozygous neighbours (Fig. 5B, C, arrowhead). These observations indicate that Dl is expressed throughout the wing anlage at low levels. The ubiquitous expression of Dl provides the explanation for the ectopic activation of the Notch pathway at an ectopic FB. Furthermore, it reveals that the effect of Fng on the activity Dl at the ectopic FB is dependent on its location: In our experimental approach, Fng is ectopically expressed in a broad stripe within the wing pouch. However, activation of Wg expression is restricted to the FB. This indicates that Dl on Fng expressing cells appears to be insufficiently active to induce expression of Wg throughout the *ptc* domain despite the presence of Fng. Induction of Wg in Fng expressing cells requires Dl expressed on Fng non-expressing PBCs. Only Dl in Fng non-expressing cells generates a signal beyond the threshold of Notch activation required to induce Wg expression in Fng expressing ABCs.

### Notch-signalling at the D/V Boundary

We asked whether we could also observe sequential activation of Notch at the D/V boundary during early wing development. Therefore, we compared the initial expression of Wg at the D/V boundary with that of Ap (Fig. 5D–G). Indeed, we found that expression of Wg is mainly restricted to VBCs (Fig. 5D–G). This observation confirms a similar observation previously made by Miccheli et al. (1997) using *wg*-lacZ and suggests that the activation of Notch is initially asymmetric also at the D/V boundary. As has been previously reported, Wg becomes expressed in dorsal and ventral boundary cells at late stages [29] (data not shown). However, we observe an asymmetry in the expression of Wg also in later stages, since expression is restricted to the row of DBCs, but occurs in VBCs (Fig. 5H, I, arrowhead) and the adjacent ventral row of cells (Fig. 5H, I, arrows).

**Figure 6:**
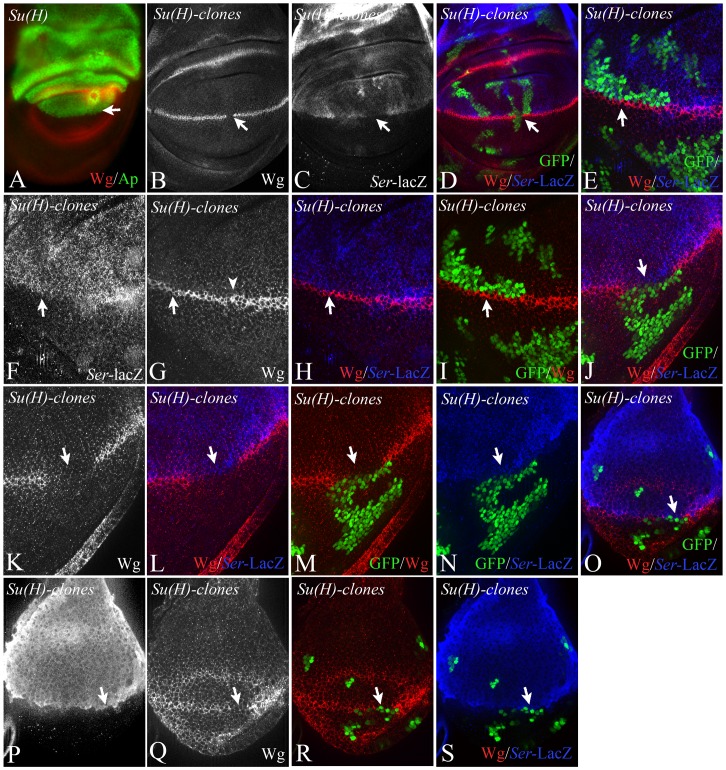
Clonal analysis of the null allele *Su(H)^d47^* with the MARCM system. Clones are labelled positively with the GFP marker. (A) Expression of Ap in *Su(H)^d47^* null mutant wing imaginal discs. The boundary between the Ap expressing and non-expressing cells (arrow) is smooth, indicating that the D/V compartment boundary has formed in the absence of *Su(H)* function. (B–P) Clones of Su(H) abutting the dorsal and/or ventral side of the D/V boundary. The dorsal cells are additionally labelled with *Ser*-lacZ. (B–D) A clone that crosses the D/V boundary causes loss of expression of Wg along the D/V boundary (arrow). (E-I) A Clone abutting the dorsal side of the D/V boundary causes a thinning, but no loss of the Wg expression domain. (J–N) In contrast, clones abutting the ventral side of the D/V boundary completely abolish expression of Wg and the vgBE (highlighted by the arrows). (O–S) A clone abutting the ventral side of the D/V boundary in an early third instar disc. Wg is interrupted in the area of the clone (arrow).

In order to investigate the importance of asymmetric Notch signalling at the D/V boundary during early wing development, we performed several experiments. First, we exploited the observation that in contrast to mutants of other genes involved in Notch signalling, *Su(H)* null mutant clones do not violate the D/V boundary [10], [27], [28] (Fig. 6A, arrow). We induced *Su(H)* clones abutting both sides of the D/V boundary. The presence of a *Ser*-lacZ construct that captures the early Ap dependent expression of Ser (construct II-9, 5 in [34]), allowed us to determine the origin of the clone. As expected, clones that cross the D/V boundary interrupted expression of Wg along the D/V boundary (Fig. 6B–D, arrow). In contrast, clones abutting the dorsal side of the boundary resulted in the restriction of Wg expression to ventral boundary cells, but did not abolish it (Fig. 6D–I). The expression of Wg was abolished in regions where mutant clones abutted the ventral side of the D/V boundary in early and late discs (Fig. 6J–S). These findings indicate that the activation of Notch in ventral boundary cells is required to establish the expression of Wg.

**Figure 7:**
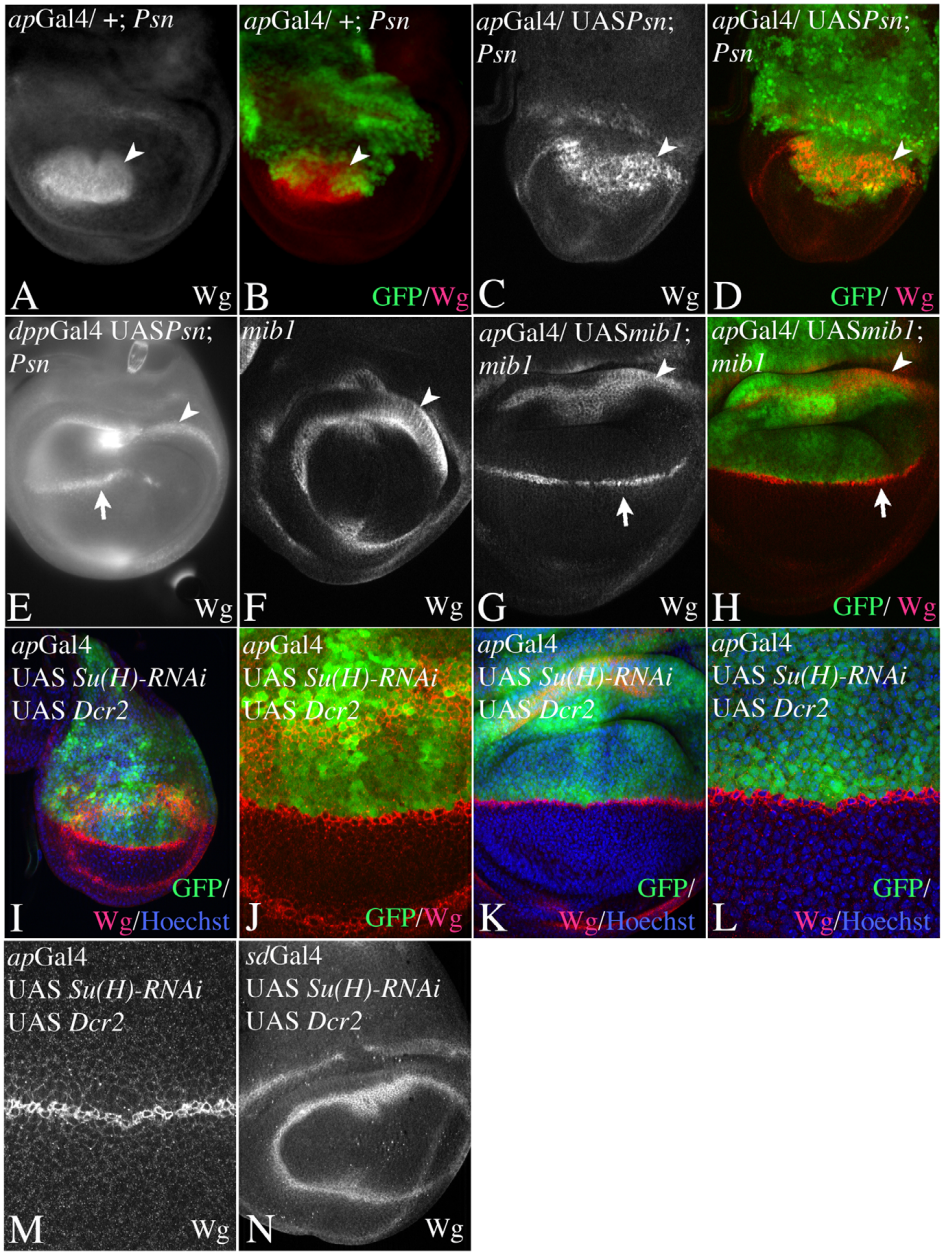
Importance of Notch activation in dorsal or ventral boundary cells for wing development. (A–D) Expression of UAS *psn* in *psn* mutant wing imaginal discs exclusively in dorsal cells with *ap*Gal4. Arrowhead points to the inner ring-like expression domain of Wg in the proximal wing anlage. (A, B) Expression of Ap (green) and Wg (red) in *Psn^C1^* mutant discs. Note that the boundary of Ap expressing and non-expressing cells is irregular because the compartment boundary fails to form. In addition the diameter of the inner ring-like domain of Wg is strongly reduced and the expression domain along the D/V boundary is lost (C, D) A similar Phenotype is observed if UAS *psn* is expressed exclusively in dorsal cells with *ap*Gal4. Thus restoring a functional Notch pathway in only dorsal cells is not sufficient to rescue expression of Wg along the D/V boundary. (E) In contrast supplying psn activity simultaneously in dorsal and ventral cells by expressing UAS Psn with *dpp*Gal4 restores Wg expression (arrow in E). (F–H) Expression of UAS mib1 in *mib1^2^*/*mib1^3^* mutant wing discs with *ap*Gal4. (F) Expression of Wg in a *mib* mutant control disc. The diameter of the residual ring-like domain of Wg expression is dramatically reduced and the expression domain along the D/V boundary is lost. (G, H) Expression of UAS *mib1* exclusively in dorsal cells with *ap*Gal4 is sufficient to restore expression of Wg along the D/V boundary (arrow) and to allow wing development to occur. (I–N) Expression of UAS *Su(H)-RNAi* during wing development with *ap*Gal4 (I–M) and *sd*Gal4 (N). Expression with *ap*Gal4 results in the formation of a nearly normal looking wing anlage. However, the comparison with the expression of Ap expression revealed that the Wg domain is restricted to VBCs in early (I, J) and late (K–M) third instar discs. (N) Expression on both half of the wing results in abolishment of expression of Wg along the D/V boundary.

Secondly, we provided Notch activity selectively only on one side of the D/V boundary (Fig. 7). We provided Notch activity exclusively in dorsal cells by expressing an UAS *Psn* construct in *Psn* null mutant discs with *ap*Gal4. We found that these discs fail to develop a proper wing anlage. Expression of Wg along the D/V boundary was absent and the diameters of the remaining ring-like expression domains of Wg were dramatically reduced as in *Psn* mutants (Fig. 7A–D). If UAS *Psn* was expressed at both sides of the boundary in *Psn* mutants, e.g. by *dpp*Gal4, expression of Wg at the D/V boundary was established (Fig. 7E, arrow). Thus, providing Notch activity exclusively in dorsal cells is not sufficient for wing development to occur.

It was not possible to exclusively express UAS *Psn* in ventral cells, because of the lack of an appropriate Gal4 line. We therefore performed alternative experiments: Firstly, we provided active Notch ligands only in dorsal cells by expression of UAS *mib1* in dorsal cells of *mib1* mutant wing discs with *ap*Gal4 (Fig. 7F–H). Previous work has demonstrated that Mib1 is absolutely required for the activity of Ser and Dl [35]–[37]. In the absence of *mib1* function, the expression of Wg along the D/V boundary is absent [35]–[37]; (Fig. 7F). Dorsally restricted expression of UAS *mib1* resulted in the establishment of Wg expression along the D/V boundary and wing development (Fig. 7G, H). Hence, a signal from dorsal to ventral boundary cells is sufficient for the initiation of expression of Wg and wing development. In the second experiment, we prevented Notch activation in dorsal cells by expression of UAS *Su(H)-RNAi* with *ap*Gal4. We found that these wing discs looked remarkably normal: the diameter of the two ring-like domains of *wg* expression is similar to wildtype and *wg* is expressed along the D/V boundary (Fig. 7I–M). A comparison with the *ap* expression domain revealed that Wg expression is restricted to VBCs also in late third instar discs (Fig. 7K–M). Hence, an active Notch pathway only in ventral cells appears to be sufficient to allow wing development to occur. Expression of UAS *Su(H)-RNAi* within DBCs and VBCs resulted in a *Su(H)* loss of function phenotype that is comparable to the null allele Su(H)^Δ47^ (Fig. 7N, compare with Fig. 6A). This result underscores the efficiency and specificity of the RNAi line. We obtained similar results by suppression of Notch activity in dorsal cells through expression of UAS *H* with *ap*Gal4 (Fig. S5A–D).

The flies depleted of *Su(H)* function developed until the adult stage and displayed the expected phenotypes. Flies where *ap*Gal4 was used had notae devoid of nearly all bristles, but had rather normal looking wings with broadened dorsal, but normal ventral wing veins and mild distal notches (Fig. S5F, G). At the margin, a few dorsal stout bristles form (not shown). This suggested the neural precursors of the dorsal bristles are induced, but probably fail to develop correctly due to the requirement of Notch throughout their lineage. This notion was confirmed by our finding that supernumerary sensory organ precursor cells developed at the dorsal side of the prospective margin in late third instar discs (Fig. S5H). This indicates that the ventrally restricted Wg domain can still pattern the wing margin in a relatively normal manner. Animals where *sd*Gal4 was used for expression had severely truncated wings that resembled that of *Ser* null mutants (Fig. S5I, compare with Fig. S3A and Fig. S5E).

**Figure 8:**
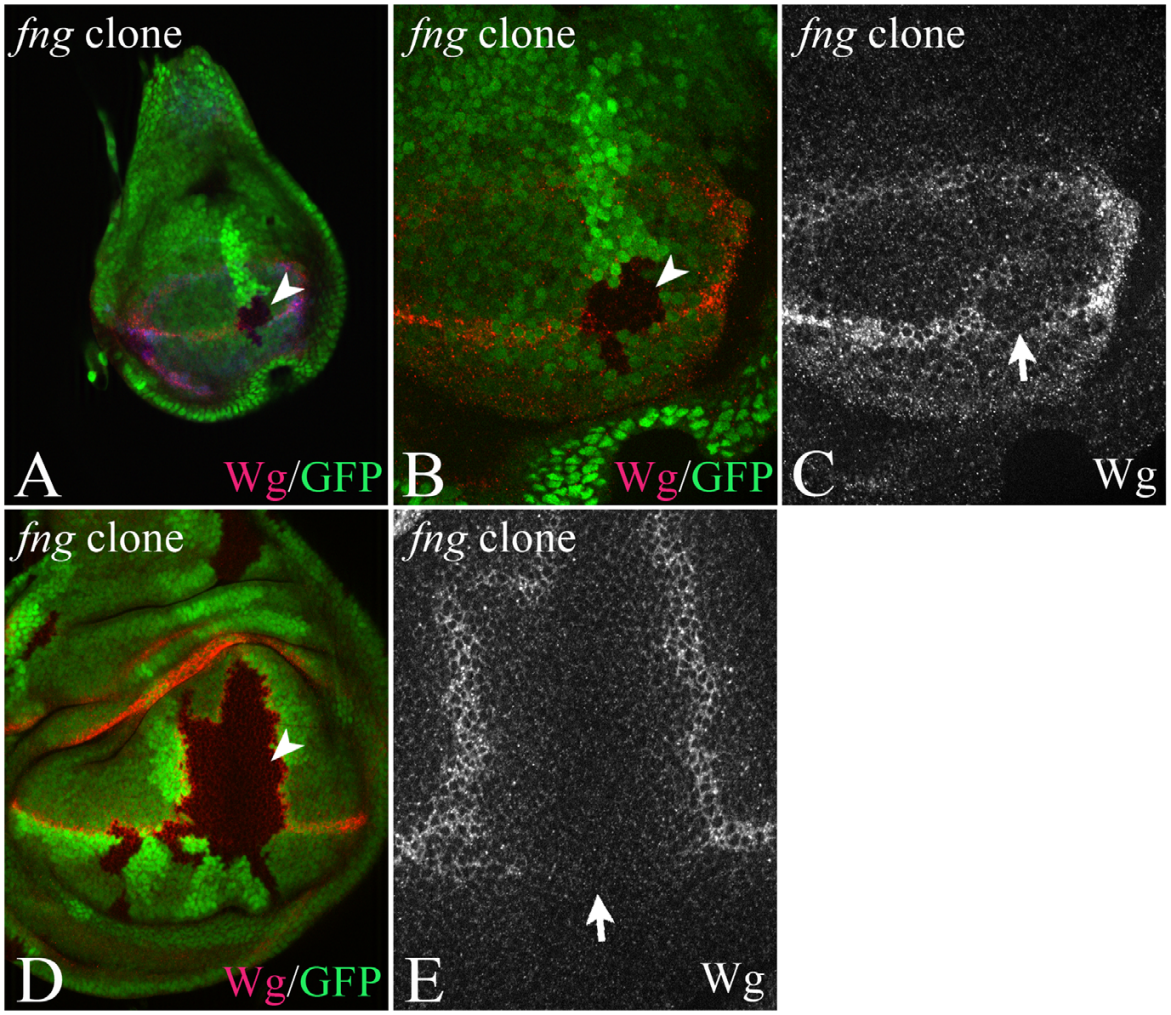
Clonal analysis of the null allele *fng^13^*. (A–E) A clone crossing the D/V boundary interrupts expression of Wg along the boundary in early (A–C) and late (D, E) third instar discs. The arrowheads point to the clone area, which is labelled by loss of the GFP marker.

We also analysed the effect of loss of *fng* function on expression of Notch target genes using clonal analysis (Fig. 8). Notably, we found that expression of Wg at the D/V boundary was interrupted in *fng*-mutant cell clones that cross the D/V boundary even during early stages of wing development (Fig. 8A–C). Apparently the loss of *wg*-expression cannot be compensated during later stages, since Wg expression was also absent in clones of older discs (Fig. 8D, E). Since Ser is required for initiation of *wg* expression in early discs, this observation suggests that Fng is required for Ser signalling to VBCs.

## Discussion

During wing development, the activity of the *Notch* pathway is required to establish a stripe-like domain of expression of several genes along the D/V boundary that control wing growth and patterning, chief among them are *wg* and *vg* [11]. The D/V boundary is a FB, which provides an interface that is crucial for the activation of Notch and establishment of this organising centre. The current understanding is that Fng promotes Dl signalling and prevents Ser signalling through the modification of Notch. As a result, Dl signals strongly from ventral to dorsal and Ser from dorsal to ventral cells boundary cells [12], [14]. The simultaneous signalling of the ligands in opposite direction establishes the expression of Notch target genes at both sides of the FB [12], [14]. It is essential for this model to work at the D/V boundary that induction of expression of Ser is restricted to dorsal and that of Dl to ventral cells. If e.g. Dl expression could also be induced in dorsal cells by the Notch pathway, the activity of Notch would immediately spread throughout the dorsal half of the wing anlage. In agreement with this requirement, it has been observed that expression of Ser is restricted to dorsal cells upon expression of activated Notch in dorsal and ventral cells [14], [19]. The combination of spatially restricted expression of the ligands and the Dl/Ser loop restricts the activation of Notch to the D/V boundary during early stages of wing development [14]. At the middle of the third larval instar stage the Dl/Ser/Wg loop takes over to maintain the activity of Notch. Thus, a critical step is the establishment of expression of Wg in boundary cells. Once this is achieved the second feedback-loop assures expression of Wg and Notch signalling throughout wing development.

While this model can explain the events at the D/V boundary, it cannot explain the events at an ectopic FB, since differential expression of the ligands is unlikely to occur there. Nevertheless, the expression of Wg is also restricted at the ectopic FB [29]. The presented work provides further evidence for the current model of Fng action, but adds new details that enable it to explain also the events at an ectopic FB. One addition is the initial sequential establishment of the expression domain of Wg through asymmetric Notch signalling during early stages of wing development. We observed asymmetric expression of Wg only in VBCs at the D/V boundary, indicating that these cells achieve sufficiently high activation of Notch to initiate expression of Wg. To us the existence of the early asymmetric phase of Notch activation was surprising given the fact that activation of Notch results in the activation of the expression of *Dl* and *Ser* [14], [15], [19]. Consequently, the activation of Notch in ventral cells should immediately lead to up-regulation of expression of Dl in ventral cells and back-signalling to dorsal cells. We therefore expected that if an asymmetric phase exists, it would be too short in time to be detected. Importantly, the initial asymmetric phase appears to be a general property for Notch signalling at a FB, since we observed on both analysed FBs. The existence of the asymmetric phase also indicates that a FB can be used to generate activity domains of the Notch pathway where the feedback-loop that regulates the expression of the ligands through Notch activation does not occur. So far the loop has been found only in the wing pouch. In the absence of the loop the asymmetric state would remain and thus, a defined stripe of high Notch activation would be generated in a field of cells that uniformly express Dl even in the absence of Ser.

At the ectopic FB, we found that eliminating the activity of the Notch pathway in PBCs does not result in the loss of expression of Wg in ABCs. It only prevents the late symmetric phase. This indicates that establishment of a Dl/Ser feedback loop in A/P boundary cells is not essential for reaching sufficient high levels of Notch signalling to induce Wg expression during the asymmetric phase. We observed the same for the D/V boundary: Depletion of *Su(H)* function or over-expression of H causes a restriction of expression of Wg to VBCs, but not its abolishment. Thus, the Dl/Ser loop is probably mainly required for the later occurring patterning of the future wing margin, but not for the establishment of the wing primordium.

A further addition is that the basic expression of Dl is independent of Ser signalling. This is indicated by the observation that 1. *Dl* is expressed throughout the wing anlage in early discs and 2. Dl signals to DBCs at the D/V boundary in absence of *Ser* function. This holds true also for the ectopic FB: here Dl signals to the ABCs in the absence of Ser. However, in both cases Dl signalling is not strong enough to initiate Wg expression, which is a crucial event for wing development.

Our results also reveal an unanticipated requirement of Ser in ABCs for the expression of Wg. This requirement could be explained through Ser signalling from the ABCs to PBCs to up-regulate Dl there, which in turn signals back to ABCs (Ser/Dl loop). This explanation would imply that the levels of Notch activation required for the induction of Dl expression are lower than that for Wg. Otherwise, the expression of Wg would not stay asymemmetric as it is observed. However, we found that Notch signalling is not required in PBCs during the early asymmetric phase. This excludes the mentioned explanation and suggests that Ser must activate Notch in the Fng expressing ABCs. This assumingly weak activation contributes to the total activity of Notch in these cells and guarantees levels of Notch signalling above the threshold required for expression of Wg. In the absence of *Ser* the activation by Dl from PBCs appears to fail to reach the threshold level in a fraction of discs. In agreement with this notion it has been shown that expression of Ser is broadly induced upon ectopic expression of Fng [14].

We further observed that concomitant loss of *Ser* and *Dl* function in ABCs always abolishes the expression of Wg at the ectopic FB. Hence, Dl must also signal in Fng expressing ABCs and contribute to Notch signalling in these cells. The results suggest that the total amount of Notch activity in ABCs is the sum of signalling by Dl and Ser in the Fng domain and Dl signalling from PBCs to ABCs. Whereby the signal from posteriors to anterior is the more important one, since its loss always abolishes expression of Wg. A similar requirement at on both sides of the boundary for Dl had been described for the D/V boundary [38]. Interestingly, our data indicate that Dl plays a similar role there as Ser at the ectopic FB.

Another addition to the current model is that Fng has two antagonistic effects on each ligand. The modification of Notch by Fringe is known to polarise signalling at the D/V-boundary by enhancing Dl- and suppressing Ser-signalling [14]. Loss of function of *fng* during early stages of wing development abolishes expression of Wg at a time where it is solely dependent on Ser signalling. This observation indicates that Fng has a positive effect on Ser signalling in addition to its known negative one: it enhances Ser-signalling from Fng expressing dorsal to non-expressing ventral cells. This enhancement is required to activate expression of Wg in cells across the boundary. This finding is in good agreement with previous work that reports that Fng can enhance the ability of Ser to induce ectopic wing margins upon their co-overexpression [39]. It is a possibility that this positive influence on Ser is indirect: The modification of Notch mediated by Fng results in a decrease of binding Ser to Notch. As a consequence high levels of free Ser are available in Fng expressing boundary cells at the FB that can bind in trans to the unmodified Notch on the adjacent Fng non-expressing boundary cells. At the analysed ectopic FB, we found that Dl is required in Fng non-expressing PBCs to raise the activity of Notch signalling to a level that is sufficient for expression of Wg in Fng expressing anterior boundary cells, although Dl is expressed ubiquitously in early discs and thus, present in both cell populations at the same levels. This finding indicates that although Dl can induce activity of Notch in the Fng domain, this activity is insufficient to initiate the expression of Wg. The activity rises beyond the threshold only if the cells receive an additional Fng-enhanced Dl signal from non-expressing. This suggests that Fng has a suppressing effect on Dl signalling within its domain. The opposing effects on the activity of each ligand have important implications for restricted Notch-signalling at the FB. Restricted expression of the ligands on opposite sides of the FB is not required to restrict Notch activation to the FB. Only Dl outside the Fng domain is sufficiently active to induce Wg expression in Fng expressing cells. In turn only Ser in Fng expressing cells is sufficiently active to induce Wg expression in Fng non-expressing cells. These conditions are only met at the FB. These properties reassure that expression of Wg is restricted to a FB even in a tissue where Dl or Ser are initially expressed uniformly, as it is the case for the ectopic FB.

A difference between the ectopic FB and the D/V boundary is that the roles of the ligands are reversed: at the ectopic FB, Dl signalling is required for the establishment of the initial asymmetric phase and Ser to establish the symmetric phase and to maintain expression of Notch activity at a high level. In contrast, Ser signalling is required for the initial asymmetric phase at the D/V boundary and Dl for the establishment of the later symmetric phase and probably maintenance during later stages. Thus, activation of Notch-signalling at a FB can be initiated by both ligands. Another difference is the location of the asymmetric stripe of *wg* expression, which is located in Fng expressing cells at the ectopic FB, but in non-expressing cells at the DV boundary. We believe that the events observed at the ectopic FB represents the more general mode of interactions, since the interactions occur solely in the ventral pouch where the cells differ mainly with respect to the expression of Fng. In contrast, at the D/V boundary dorsal cells differ from ventral cells by expression the selector Ap, which might modify the outcome of the interactions.

### Interactions at a Boundary of Fng Expressing and Non-expressing Cells

On the basis of our results, we first summarise the events at the ectopic boundary. During early stages of wing development, Dl is ubiquitously expressed throughout the wing anlage. Ectopic expression of *fng* with *ptc*Gal4, creates a band-like Fng domain in the ventral pouch. During early stages Dl and Ser (probably induced by Dl) activate Notch signalling throughout the Fng domain at low levels that are not sufficient for activation of Wg. At the sharp posterior FB, Dl signalling from PBCs to ABCs, enhanced by Fng, raises the levels of Notch in ABCs beyond the threshold required for expression of Wg. The asymmetric phase is established. Dl signalling also up-regulates expression of Ser in ABCs. Over time *Ser* signalling from ABCs to PBCs (enhanced by Fng), induces Wg expression in PBCs. After the solid induction of symmetric expression of Wg, the Dl/Ser/Wg loop takes over and maintains Notch signalling at the D/V boundary. We observed that the expression of Gbe+Su(H)-lacZ is broader upon ectopic expression of Fng in *Ser* mutant early third instar discs. Moreover, Wg is ectopically expressed throughout the ventral *ptc* domain upon ectopic expression of Fng by with *ptc*Gal4 in *Ser H*, but not in *H* mutant discs. Thus, Ser probably contributes to keeping the Notch activity in the Fng domain at low level. It is known that the expression of Ser can contribute to the suppression of Notch activity in a cell-autonomous manner through cis-inhibition [40]. Cis-inhibition has been discovered during analysis of wing development and appears to be involved in regulation and directional Notch signalling in several processes (Summarised in [41]. This mechanism causes strong Ser signalling only from Ser expressing to non-expressing cells. Since Ser expression is induced in the Fng domain, strong signalling occurs from ABCs to PBCs. Thus, it is likely that cis-inhibition contributes to the directional signalling of from ABCs to PBCs to induce the later symmetric phase.

At the D/V boundary signalling is initiated differently. We have shown here that loss of the activity of the Notch pathway in dorsal cells does not prevent the establishment of expression of Wg along the D/V boundary, but restricts it to VBCs and allows wing development to proceed. Thus, the dorsal to ventral signal, which is mediated by Ser is most important. This notion is also in agreement with the null phenotype of *Ser* (this work and [30]). Ser and Fng are initially expressed in all dorsal cells [20]. Fng enhances Ser signalling to VBCs, but suppresses signalling among dorsal cells. This strong polarised signalling results in the activation of Wg expression and up-regulation of Dl expression in VBCs (Fig. 7d). It is likely, that the cis-inhibitory effect of Ser [40] contributes to the suppression of the activation of Notch in dorsal cells through the initial phase of wing development, since we observe the expansion of the expression of Gbe+Su(H)–lacZ over the whole dorsal wing anlage in early *Ser* mutant discs. This is probably induced by the weak ubiquitous expression of Dl we observed in early wing discs. The analysis of the *Ser* null and *Ser H* double mutants indicates that Dl can activate Notch signalling in absence of *Ser* function, but not strong enough to induce Wg expression, despite the presence of the FB. It appears that at the D/V boundary, Ser has to up-regulate the expression of Dl in VBCs over time beyond the threshold that is required to induce Wg expression in DBCs. Fng contributes to induction of Wg expression by enhancing Dl signalling from Fng non-expressing VBCs to expressing DBCs. The requirement for accumulation of Dl could contribute to the observed delay of the establishment of the symmetric phase of expression. After the initial asymmetric phase, the Ser/Dl/Wg loop is established to maintain Notch signalling and symmetric expression of Wg. Our results indicate that the initial Fng enhanced *Ser* signal is sufficiently strong to induce Wg in VBCs in a manner that enables also the establishment of the Ser/Dl/Wg loop. This is indicated by the observation that suppression of Notch activity in all dorsal cells throughout wing development does not prevent maintenance of expression of Wg in late stages of the third instar and even allows the development of adult wings with only minor patterning problems. Thus, the later signal from VBCs to DBCs is mainly required for positioning and patterning of the wing margin.

Asymmetric signalling through the Hh pathway has been shown to establish the organising centre for the A/P axis at the anterior side of the boundary ([42]). It has been assumed that one difference in the establishment of the D/V organising centre is its symmetric placement on the D/V boundary. Our results suggest that at least during initial phases, the signalling at the D/V boundary is also asymmetric and that the established organising centre can also work if it is displaced ventrally. Thus, it appears that the signalling events at both boundaries are more similar than previously anticipated. We have previously observed that also mechanism for the establishment of both corresponding compartment boundaries is more similar than anticipated [10].

## Materials and Methods

### Fly Stocks

*fng^13^* FRT80 [43], *Dl^rev10^* FRT82B and *Ser^VX82^* FRT82B [16], *Psn^C1^* FRT2A [44], *mib1^1^* and *mib1^3^* [36], *Su(H)^d47^ P(B)* FRT40A [45], *Ser^RX106^* is a 9k b deletion within the transcription unit that leads to a complete loss of expression [46], *H^E31^* [47], *ap^UG035^* [48], Gbe+Su(H)-lacZ [31], *vg*BE-lacZ [49], *Ser*-lacZ [50].

UAS *fng* [29], UAS *Flp* [51], UAS *vg* [21], UAS *Ser* [30], UAS *Dl* [19] *UAS H* [7], UAS *mib1* [36], *and* UAS *GFP* [52], *UAS GFP-GPi* [53], UAS *Su(H)-RNAi* (DGRC 3497R-1), UAS *Ser-RNAi* (VDRC 27172), UAS *Dcr2* [54]. The constructs were used in combination with *sd*Gal4, *ptc*Gal4, *dpp*Gal4, and *ap*Gal4.

### Clonal Analysis

Clones were induced with an *hs*FLP construct during the first larval instar (24–48 h after egg laying).

### Histochemistry

Following antibodies have been used: αNotch raised against the extracellular domain, αβ-Gal, αWg, αDl and αSer. The αNotch, αWg, αDl and αHnt antibodies were obtained from the Developmental Studies Hybridoma Bank developed under the auspices of the NICHD and maintained by the University of Iowa, Department of Biological Sciences, Iowa City, IA 52242. αβ-Gal was purchased from Cappel. αSer was a gift of E. Knust [46]. Staining was performed according to standard protocols. FITC, Alexa 488, Alexa 568, Alexa 647 and Texas Red conjugated secondary antibodies were purchased from Jackson Immuno Research and Invitrogen.

## Supporting Information

Figure S1 (A–C) Expression of ptcGal4 and Wg throughout the third larval instar stage. (A) Wg is initially expressed in a ventral domain that defines the wing anlage. (B) This ventral domain results in a stripe-like domain along the D/V boundary (arrow) and a proximal ring-like domain (arrowhead). At late stages a second ring-like domain appears (arrowheads). *ptc*Gal4 (green) is always expressed in a band-like domain in the centre of the wing anlage. (D–F) Expression of Dl in a wildtype disc (D, E) and a disc where *fng* is ectopically expressed with ptcGal4. The late expression pattern of Dl is shown. (D, E) Dl is expressed in two bands adjacent to the D/V boundary which is revealed in (E) through the expression of the notch target Gbe+Su(H)-lacZ. Similar bands are recognisable in the region of ectopic FB (arrowheads in F).
(TIF)

Figure S2 Range of the ectopic Wg expression domain induced by *ptc*Gal4 UAS *fng* in different genetic backgrounds. (A-C) Early third instar discs. (D-F) Late third instar discs. For further explanation, see Text S1.
(TIF)

Figure S3 The Analysis of *Ser^RX106^* mutants.(A, A’) *Ser^RX106^* mutant flies lack a wing and halteres. They bear either a small wing rudiment (A) or a wing to notum duplication (A’). (B–D, G, H) Expression of Wg and Gbe+Su(H)-lacZ in a late (B–D) and early (G, H) third larval instar wing imaginal disc. (E, F) Expression of Wg in *Ser^RX106^* mutant wing discs. The arrows highlight the area of the wing anlage. In mutant discs either the outer ring-like domain of Wg is left (E) or a second band-like domain characteristic for the notum can be observed (F). Expression of Wg along the D/V boundary (arrows in G–I) is absent in early third instar disc mutant for *Ser^RX106^* (G–J). However, expression of Gbe+Su(H)-lacZ is present (see arrows in G–I, K). (M–Q) This situation is maintained also in later discs. (P, Q) A *Ser^RX106^* mutant discs of the mid third instar at higher magnification showing expression of Gbe+Su(H)-lacZ. Arrowheads in (P, Q) mark the extents of the dorsal side of the rudimentary anlage. Note, that the expression of Gbe+Su(H)-lacZ in dorsal cells disappears in late discs (see panel (T)). (R–T) Expression of UAS *fng* in *Ser^RX106^* mutant wing discs during early third instar with *ptc*Gal4 results in the ectopic expression of Gbe+Su(H)-lacZ (arrow in R, S). Note, that the expression of Gbe+Su(H)-lacZ is restricted to the *ptc* domain. (T) The ectopic expression of Gbe+Su(H)-lacZ is lost during later stages of development.
(TIF)

Figure S4 Analysis of the *Ser^RX106^H^E31^* double mutants.(A, A’) Pharate adults have halteres and wings with a proper margin, indicated by the innervated bristles characteristic for the margin (shown in higher magnification in (A’)). (B-I) Analysis of *Ser^RX106^H^E31^* double mutant wing imaginal discs. Expression of Gbe+Su(H)-lacZ (B), Wg (C) along the D/V boundary. (D) Expression of the two Notch targets in relation to that of Ap. The magnification of the region highlighted with the arrow in (D) accentuates the fact that expression of the Notch targets is restricted to Ap-expressing DBCs (E). (F) Expression of Gbe+Su(H)-lacZ at the D/V boundary is extinguished in *ap^UG035^Ser^RX106^H^E31^* triple mutant wing imaginal discs. (G) Expression of Dl in *Ser^RX106^H^E31^* double mutant wing imaginal discs. (H, I) Expression of Gbe+Su(H) and Ap in a early third instar *Ser^RX106^H^E31^* double mutant wing imaginal disc. Expression of Gbe+Su(H)-lacZ is expands throughout the Ap-expressing dorsal half of the disc (arrows). (J-N) Expression of UAS *fng* in *Ser^RX106^H^E31^* double mutant wing imaginal discs results in the ectopic expression of Gbe+Su(H)-lacZ (J) and Wg (L) (arrow in J–M). The merge of (J–L) reveals that the ectopic expression of the marker is restricted to the *ptc* domain, even in late third instar. The expression of the markers in PBCs seen in wt and *H^E31^* mutant discs (arrow in N) is missing. Note that the expression of Wg can be observed in a broad band throughout the *ptc* domain and is not restricted to the FB as in the wildtype.
(TIF)

Figure S5 (A-D) Expression of UAS *H* with *sd*Gal4 (A) and *ap*Gal4 (B–D). Expression with *sd*Gal4 results in a loss of Wg expression along the D/V boundary, while the expression with *ap*Gal4 restricts the expression along the D/V boundary to VBCs (arrow in B–D). (E) Expression of UAS *Ser-RNAi* only in dorsal wing cells with *ap*Gal4 results in a severe truncation of the wing (arrow). (F, G) In contrast, expression of UAS *Su(H)-RNAi* in the same manner allows the formation of a nearly normal wing with broadened dorsal wing veins (arrow). (H) Wing disc of a fly where UAS *Su(H)-RNAi* was expressed with *ap*Gal4. The arrow points to the supernumerary sensory organ precursor cells formed on the dorsal side of the wing pouch in the absence of Su(H). (I) Expression of UAS *Su(H)-RNAi* in dorsal and ventral cells results in a severe truncation of the wing (arrow).
(TIF)

Text S1 The phenotype of *Ser^RX106^* flies.
(DOCX)
