# Supplementary figures and images for: Sequential Notch Signalling at the Boundary of Fringe Expressing and Non-Expressing Cells
Source: PLoS One. 2012 Nov 12;7(11):e49007. doi: 10.1371/journal.pone.0049007 (PMC3495781; doi:10.1371/journal.pone.0049007)

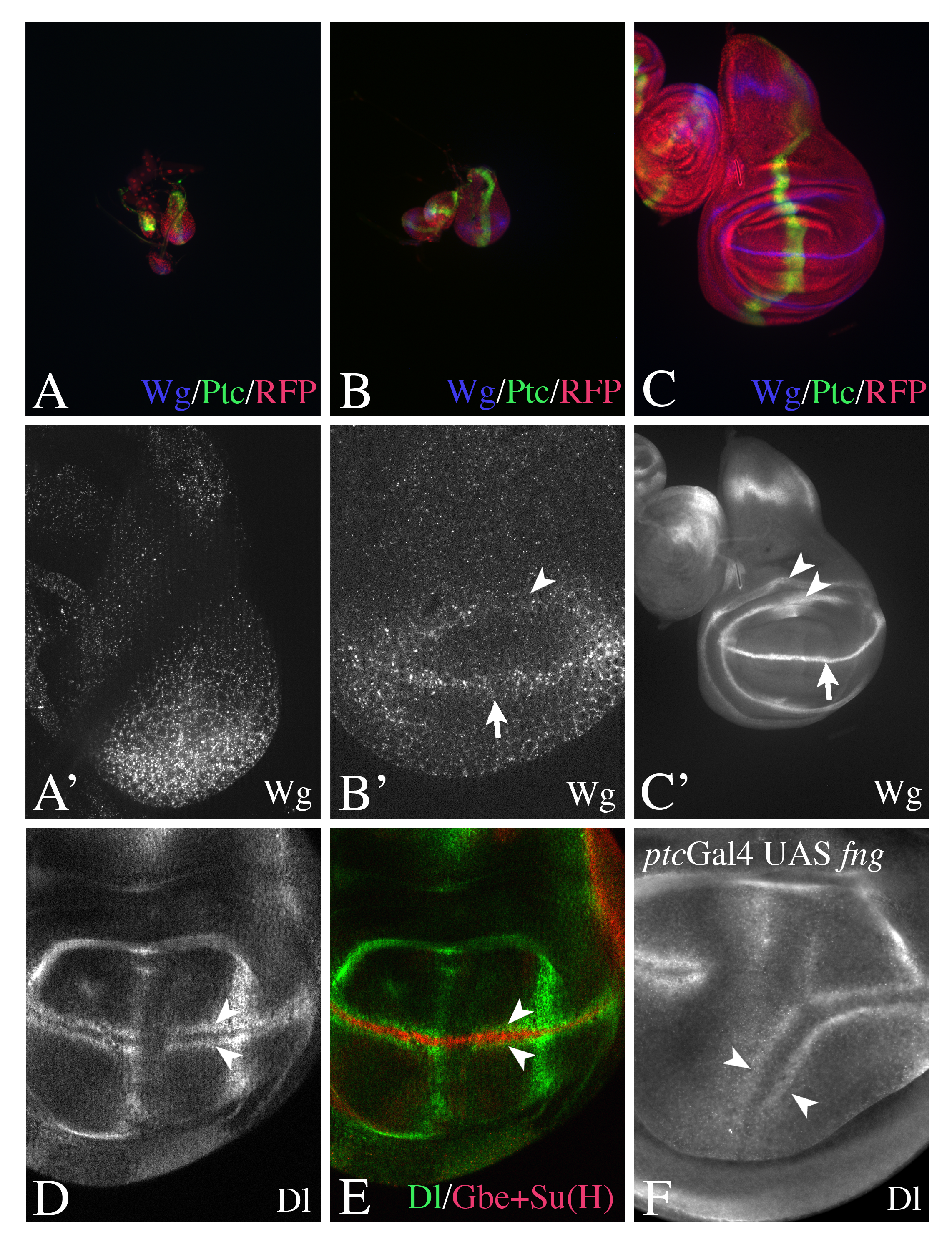

Supplement: Figure S1 — (A–C) Expression of ptcGal4 and Wg throughout the third larval instar stage. (A) Wg is initially expressed in a ventral domain that defines the wing anlage. (B) This ventral domain results in a stripe-like domain along the D/V boundary (arrow) and a proximal ring-like domain (arrowhead). At late stages a second ring-like domain appears (arrowheads). ptcGal4 (green) is always expressed in a band-like domain in the centre of the wing anlage. (D–F) Expression of Dl in a wildtype disc (D, E) and a disc where fng is ectopically expressed with ptcGal4. The late expression pattern of Dl is shown. (D, E) Dl is expressed in two bands adjacent to the D/V boundary which is revealed in (E) through the expression of the notch target Gbe+Su(H)-lacZ. Similar bands are recognisable in the region of ectopic FB (arrowheads in F). (TIF) [file pone.0049007.s001.tif]

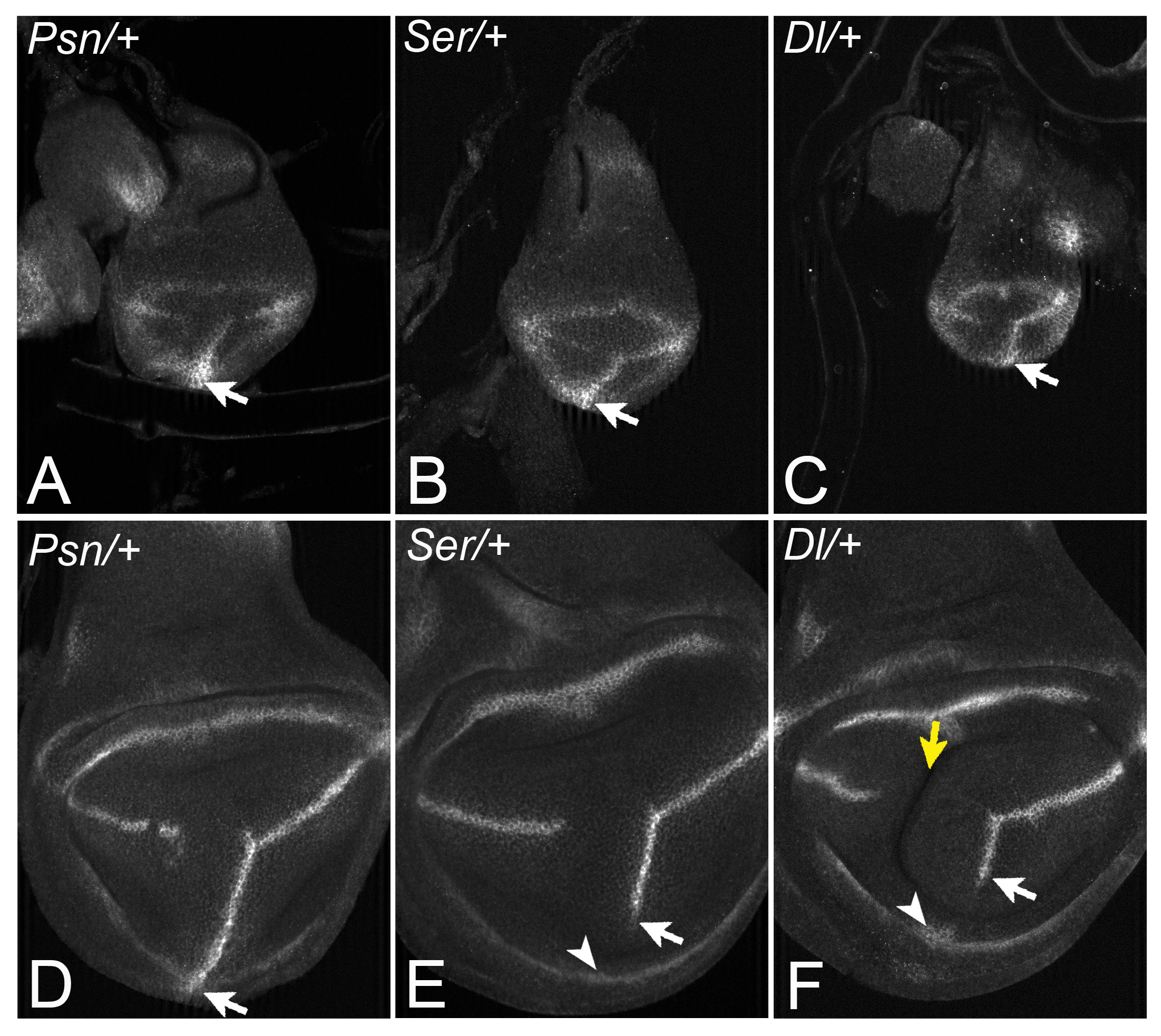

Supplement: Figure S2 — Range of the ectopic Wg expression domain induced by ptcGal4 UAS fng in different genetic backgrounds. (A-C) Early third instar discs. (D-F) Late third instar discs. For further explanation, see Text S1. (TIF) [file pone.0049007.s002.tif]

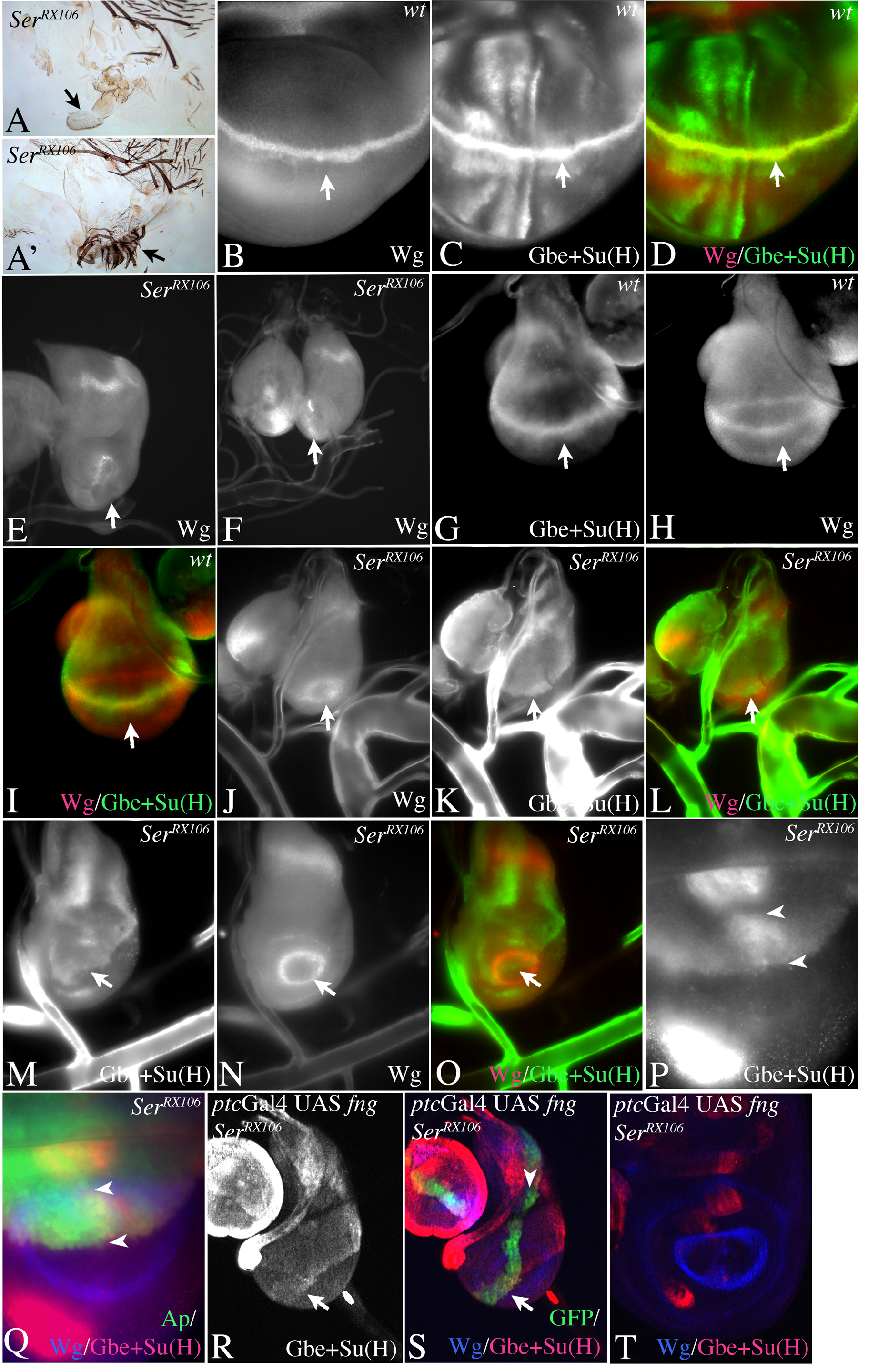

Supplement: Figure S3 — The Analysis of SerRX106 mutants.(A, A’) SerRX106 mutant flies lack a wing and halteres. They bear either a small wing rudiment (A) or a wing to notum duplication (A’). (B–D, G, H) Expression of Wg and Gbe+Su(H)-lacZ in a late (B–D) and early (G, H) third larval instar wing imaginal disc. (E, F) Expression of Wg in SerRX106 mutant wing discs. The arrows highlight the area of the wing anlage. In mutant discs either the outer ring-like domain of Wg is left (E) or a second band-like domain characteristic for the notum can be observed (F). Expression of Wg along the D/V boundary (arrows in G–I) is absent in early third instar disc mutant for SerRX106 (G–J). However, expression of Gbe+Su(H)-lacZ is present (see arrows in G–I, K). (M–Q) This situation is maintained also in later discs. (P, Q) A SerRX106 mutant discs of the mid third instar at higher magnification showing expression of Gbe+Su(H)-lacZ. Arrowheads in (P, Q) mark the extents of the dorsal side of the rudimentary anlage. Note, that the expression of Gbe+Su(H)-lacZ in dorsal cells disappears in late discs (see panel (T)). (R–T) Expression of UAS fng in SerRX106 mutant wing discs during early third instar with ptcGal4 results in the ectopic expression of Gbe+Su(H)-lacZ (arrow in R, S). Note, that the expression of Gbe+Su(H)-lacZ is restricted to the ptc domain. (T) The ectopic expression of Gbe+Su(H)-lacZ is lost during later stages of development. (TIF) [file pone.0049007.s003.tif]

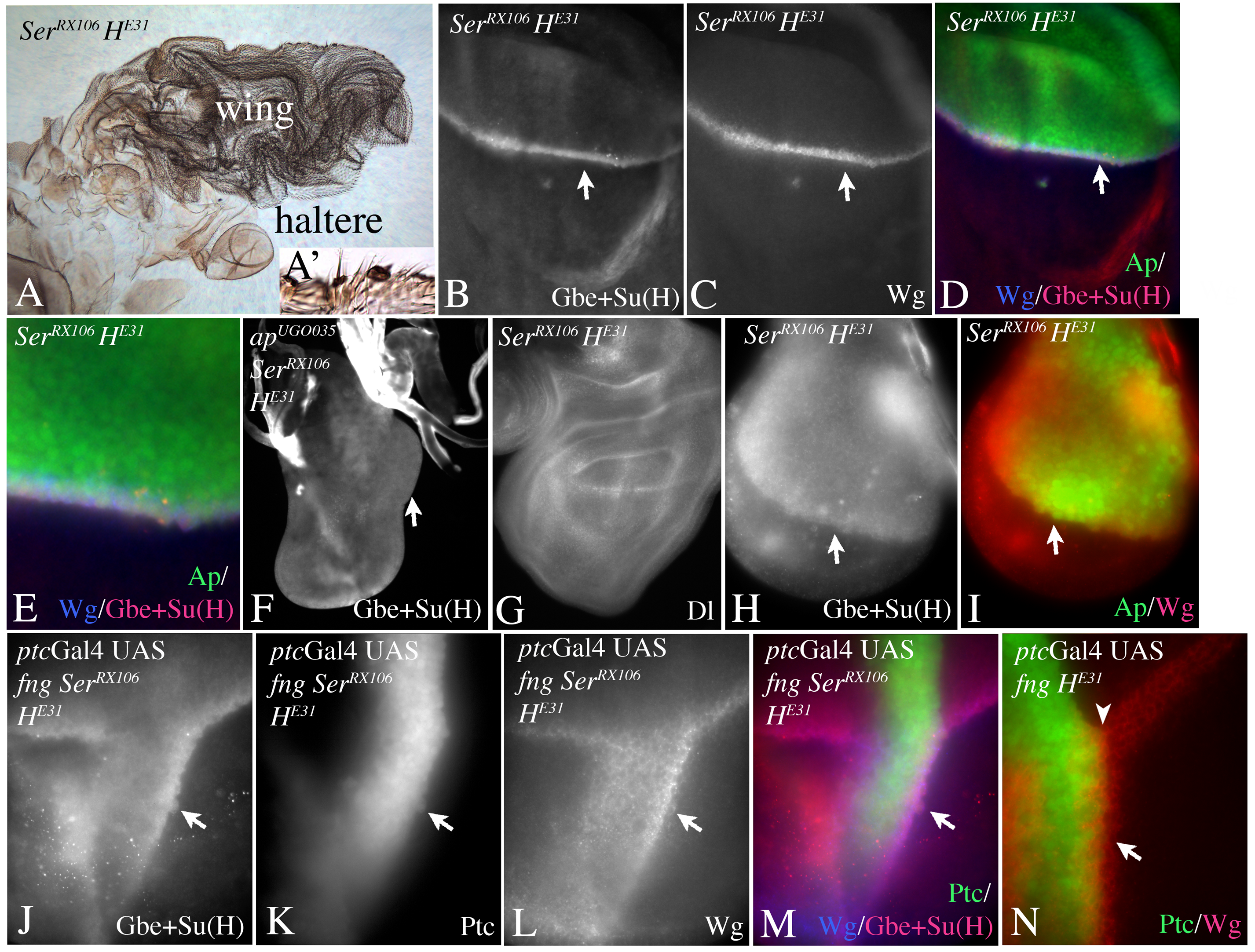

Supplement: Figure S4 — Analysis of the SerRX106HE31 double mutants.(A, A’) Pharate adults have halteres and wings with a proper margin, indicated by the innervated bristles characteristic for the margin (shown in higher magnification in (A’)). (B-I) Analysis of SerRX106HE31 double mutant wing imaginal discs. Expression of Gbe+Su(H)-lacZ (B), Wg (C) along the D/V boundary. (D) Expression of the two Notch targets in relation to that of Ap. The magnification of the region highlighted with the arrow in (D) accentuates the fact that expression of the Notch targets is restricted to Ap-expressing DBCs (E). (F) Expression of Gbe+Su(H)-lacZ at the D/V boundary is extinguished in apUG035SerRX106HE31 triple mutant wing imaginal discs. (G) Expression of Dl in SerRX106HE31 double mutant wing imaginal discs. (H, I) Expression of Gbe+Su(H) and Ap in a early third instar SerRX106HE31 double mutant wing imaginal disc. Expression of Gbe+Su(H)-lacZ is expands throughout the Ap-expressing dorsal half of the disc (arrows). (J-N) Expression of UAS fng in SerRX106HE31 double mutant wing imaginal discs results in the ectopic expression of Gbe+Su(H)-lacZ (J) and Wg (L) (arrow in J–M). The merge of (J–L) reveals that the ectopic expression of the marker is restricted to the ptc domain, even in late third instar. The expression of the markers in PBCs seen in wt and HE31 mutant discs (arrow in N) is missing. Note that the expression of Wg can be observed in a broad band throughout the ptc domain and is not restricted to the FB as in the wildtype. (TIF) [file pone.0049007.s004.tif]

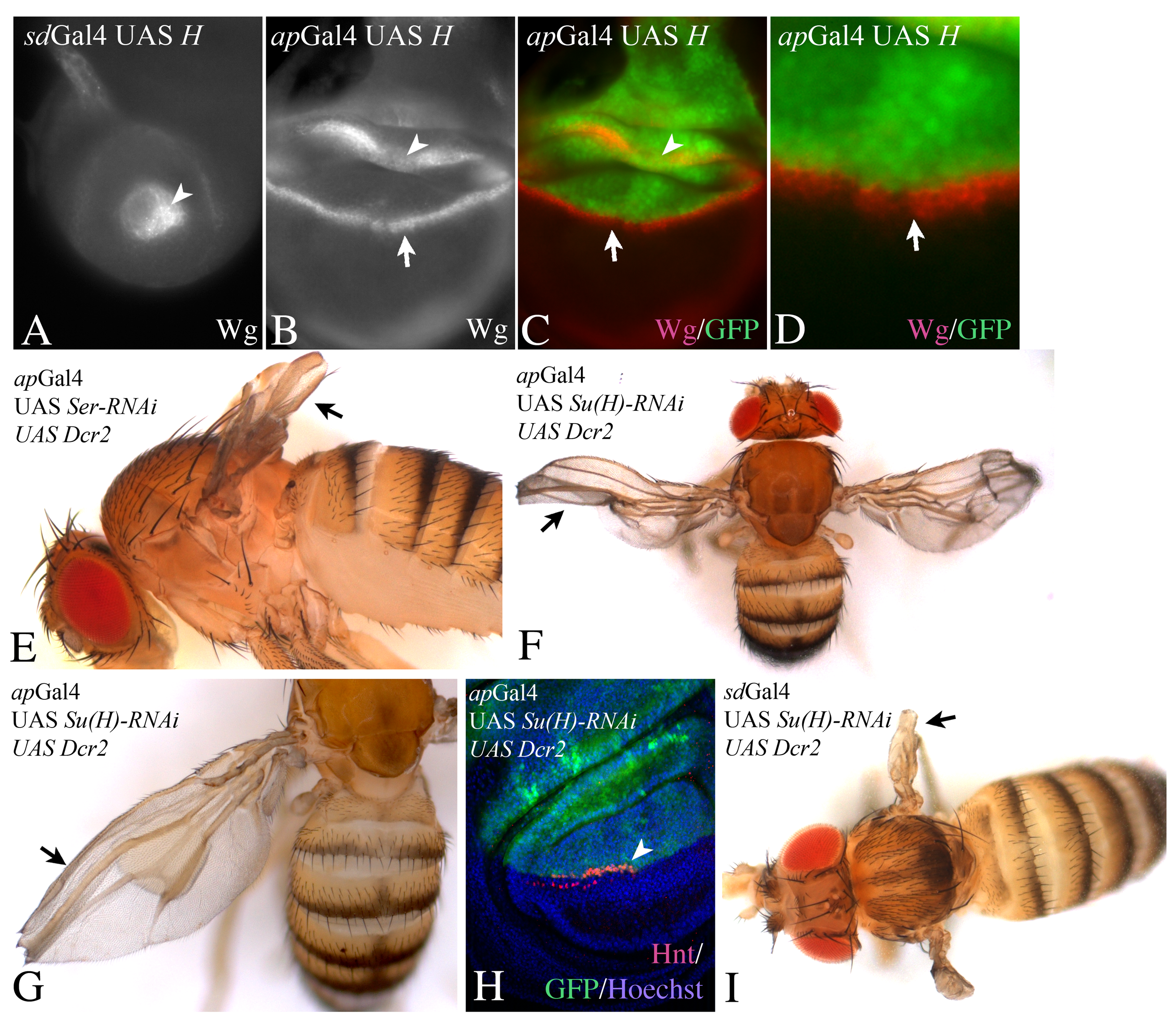

Supplement: Figure S5 — (A-D) Expression of UAS H with sdGal4 (A) and apGal4 (B–D). Expression with sdGal4 results in a loss of Wg expression along the D/V boundary, while the expression with apGal4 restricts the expression along the D/V boundary to VBCs (arrow in B–D). (E) Expression of UAS Ser-RNAi only in dorsal wing cells with apGal4 results in a severe truncation of the wing (arrow). (F, G) In contrast, expression of UAS Su(H)-RNAi in the same manner allows the formation of a nearly normal wing with broadened dorsal wing veins (arrow). (H) Wing disc of a fly where UAS Su(H)-RNAi was expressed with apGal4. The arrow points to the supernumerary sensory organ precursor cells formed on the dorsal side of the wing pouch in the absence of Su(H). (I) Expression of UAS Su(H)-RNAi in dorsal and ventral cells results in a severe truncation of the wing (arrow). (TIF) [file pone.0049007.s005.tif]
