# Supplementary material for: Sequential Notch Signalling at the Boundary of Fringe Expressing and Non-Expressing Cells
Source: PLoS One. 2012 Nov 12;7(11):e49007. doi: 10.1371/journal.pone.0049007 (PMC3495781; doi:10.1371/journal.pone.0049007)
Supplement: Text S1 — The phenotype of SerRX106 flies. (DOCX) [file pone.0049007.s006.docx]

**Text S1: The phenotype of *Ser^RX106^* flies**

Some of the *Ser^RX106^*homozygous flies develop to the pharate adult stage. These escapers exhibited a range of phenotypes typically for *Ser* mutants, such as missing joints between the tarsal segments of the legs, wing rudiments and missing halteres ([[30](#_ENREF_30)], Fig. S3A, A’). In addition to the reported defects, we observed that the joint between the femur and tibia is absent and both segments are fused along their longitudinal axis (data not shown). These defects are also seen upon loss of function of Notch activity [[53](#_ENREF_53),[54](#_ENREF_54)]. Some of the mutant flies had a stronger truncation of the wing than previously reported and in a small number of flies the wing was replaced by a small duplication of the notum (Fig. S3A’). The observed adult phenotypes were already recognisable in wing imaginal discs (Fig. S3E, F, N): Using the late expression pattern of Wg as marker, we found that the largest fraction displayed the phenotype described before: the expression of *wg* along the D/V boundary was absent and the diameters of the two ring-like domains in the anlagen of the proximal wing are strongly reduced (Fig. S3B, N). These defects indicate a truncation of the proximo-distal (P/D) axis of the wing from the distal part of the proximal wing onwards. In addition, we found discs, with only the outer ring-like expression domain of *wg* present, indicating an even stronger truncation of the wing (Fig. S3E, arrow). In a small fraction of discs all expression domains of *wg* characteristic for the wing were lost and replaced by a second stripe-like domain characteristic for the notum (Fig. S3F, arrow). This phenotype corresponds to the wing to notum transformation observed in a low percentage of the pharate adults (Fig. S3A’). Nevertheless, the average phenotype of *Ser^RX106^* null-mutants is weaker than that of mutants of other genes required for *Notch* signalling, e. g. *Psn*, or *nicastrin* mutants (e. g. see [[10](#_ENREF_10)]). One explanation for this discrepancy is that residual activity of the *Notch* pathway exists, even in the complete absence of *Ser* function as we have suggested previously [[10](#_ENREF_10),[19](#_ENREF_19)]. Interestingly, the loss of *Ser* function has little effect on compartment formation, as documented by the smooth boundary between *ap* expressing and non-expressing cells in *Ser* mutant wing discs (Fig. S3Q). Thus, D/V compartment formation appears to require less activity of the Notch pathway than wing development.
